# Gestational Valproate Alters BOLD Activation in Response to Complex Social and Primary Sensory Stimuli

**DOI:** 10.1371/journal.pone.0037313

**Published:** 2012-05-17

**Authors:** Ada C. Felix-Ortiz, Marcelo Febo

**Affiliations:** 1 Department of Psychology, Northeastern University, Boston, Massachusetts, United States of America; 2 Department of Psychiatry, The McKnight Brain Institute, University of Florida, Gainesville, Florida, United States of America; Tokai University, Japan

## Abstract

Valproic acid (VPA) has been used clinically as an anticonvulsant medication during pregnancy; however, it poses a neurodevelopmental risk due to its high teratogenicity. We hypothesized that midgestational (GD) exposure to VPA will lead to lasting deficits in social behavior and the processing of social stimuli. To test this, animals were given a single IP injection of 600 mg/kg of VPA on GD 12.5. Starting on postnatal day 2 (PND2), animals were examined for physical and behavior abnormalities. Functional MRI studies were carried out after PND60. VPA and control animals were given vehicle or a central infusion of a V_1a_ antagonist 90 minutes before imaging. During imaging sessions, rats were presented with a juvenile test male followed by a primary visual stimulus (2 Hz pulsed light) to examine the effects of prenatal VPA on neural processing. VPA rats showed greater increases in BOLD signal response to the social stimulus compared to controls in the temporal cortex, thalamus, midbrain and the hypothalamus. Blocking the V_1a_ receptor reduced the BOLD response in VPA animals only. Neural responses to the visual stimulus, however, were lower in VPA animals. Blockade with the V_1a_ antagonist did not revert this latter effect. Our data suggest that prenatal VPA affects the processing of social stimuli and perhaps social memory, partly through a mechanism that may involve vasopressin V_1a_ neurotransmission.

## Introduction

Autism spectrum disorders (ASD), which includes autism, Asperger's syndrome, and pervasive developmental disorder not otherwise specified (PD-NOS), are characterized by deficits in verbal and non-verbal communication, reduced social interactions, and restricted range of interests and motor stereotypies. Genome-wide screening has identified numerous gene mutations that might underlie ASD [1]. Among the candidate genes for ASD are those encoding the arginine-vasopressin (AVP) V_1a_ receptor (V1AR) [2] and the oxytocin receptor [1], which are known neural substrates that modulate the expression of social behavior [3], [4]. However, a wider range of genes are linked to ASD, which supports the notion of multiple developmental origins. Despite our growing knowledge of the genes associated with ASD, research is needed in order to establish animal models that accurately depict its underlying neurobiology and multiple origins. Developmental insults during specific stages of CNS development contribute to the etiology of ASD. Medications treating depression, hypertension and epilepsy during pregnancy may increase the risk of fetal malformations and ASD [5], [6], [7], [8]. These include valproic acid (VPA), carbamazepine, thalidomide, and phenytoin [9]. Of all the aforementioned drugs, only VPA results in similar birth defects in rats and has provided a useful tool to model ASD [9], [10], [11]. VPA, which is commercially available as Depakote®, is a mood stabilizer and anticonvulsant, given during pregnancy to women suffering from epilepsy. Fetal exposure at embryonic day E20–E24 can produce neural tube defects and ASD. In rats, the teratologic effects similar to those reported in humans are observed when exposure occurs between E9.5–12.5 [9], [12], [13], [14]. Reports indicate that VPA treated rats show early signs of developmental abnormalities. These include a longer latency for eye opening compared to healthy pups, deficits in olfactory discrimination in the nest, and problems with motor performance as early as postnatal days 8–9 (PD8–9) [10]. Deficits in fear condition [15], eye-blink conditioned reflex [14], and motor stereotypies have also been reported [16] and are consistent with the notion that VPA produces motor and cognitive behavioral features in rats partially resembling ASD. Abnormalities in size of cerebellar [17] and brainstem auditory nuclei [9] and of motor [14] and sensory cortical neuron morphology [18] have been reported in rats treated prenatally with VPA, which parallels postmortem pathological findings in autistics. These data lends support to the use of the VPA rat model in studies of the neurobiology of ASD.

Despite the growing amount of experiments on the effects of gestational VPA on neurodevelopment, a detailed investigation of social neural processing is yet to be carried out. Here, we tested whether prenatally exposed rats show abnormal behavioral and neurophysiological responses to social and non-social (visual) stimuli. The experiments were designed to track the developmental progress of various social behaviors from the early postnatal period to adulthood. Given the available evidence cited above, we anticipated that gestational VPA would dramatically alter the way the specific brain regions of the rat respond to a social stimulus. Moreover, the role of vasopressin in modulating the neural response to a social stimulus was assessed in adult rats exposed prenatally to VPA. Genes encoding the vasopressin (AVP) V_1a_ receptor have been linked to ASD [2]. Within the mammalian central nervous system, the modulatory actions of AVP are predominantly mediated by the V_1a_ receptor subtype, which is distributed throughout several limbic subcortical nuclei in rodent brain [19]. Recent evidence indicates that neonatal VPA exposure can influence vasopressin immunolabelling in several regions of the rodent brain such as the anterior hypothalamic area and mediodorsal thalamus, and influences olfactory-based social behavior in rodents [20]. Therefore, it was also hypothesized here that the role of V_1a_ mediated neurotransmission in the neural processing of a social stimulus would be altered by gestational exposure to VPA.

## Results

**Figure 1:**
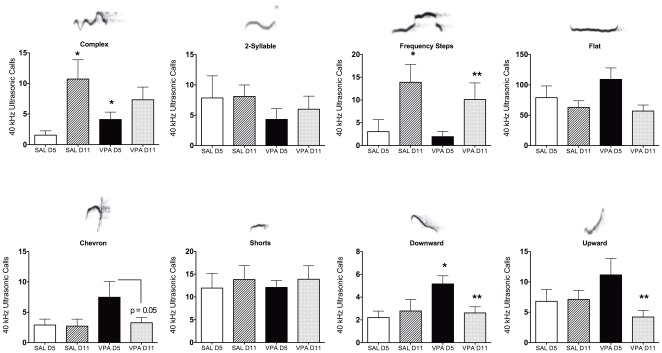
Number of ultrasonic vocalizations (USV) emitted by saline (SAL) and valproic acid (VPA) pre-exposed pups. Data were collected on postnatal days 5 and 11 (D5 and D11). USV's were categorized and analyzed separately according to specific USV characteristics. Bar graphs all have representative sonograms overlying the category title. All data presented as mean ± standard error. *Significantly different from SAL D5; **significantly different from VPA D5 (Kruskall-Wallis Analysis of Variance, p<0.05). VPA D11 showed a nonsignificant trend when compared to VPA D5 (p = 0.05).

**Figure 2:**
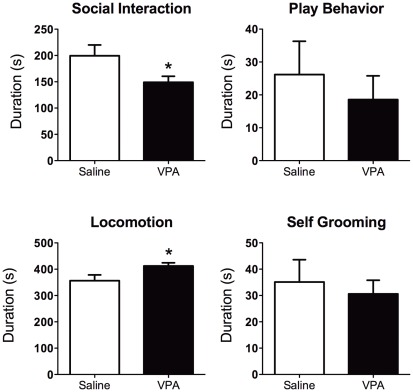
Behavior of adolescent rats pre-exposed to saline or valproic acid (VPA) during gestation. Animals were tested for social interactions, play behaviors, spontaneous locomotor activity, and self-grooming. Rats showed lower social interaction (t-test t_20_ = 2.1, p = 0.04) and greater locomotor activity (t-test t_20_ = 2.1, p = 0.03). No differences were observed with play behavior and self-grooming. However, play attacks to the nape were significantly lower in VPA compared with SAL animals (t_20_ = 2.1, p = 0.04; data not shown). All data shown as mean ± standard error. *Significantly different from SAL (two-tailed t-test, p<0.05).

**Figure 3:**
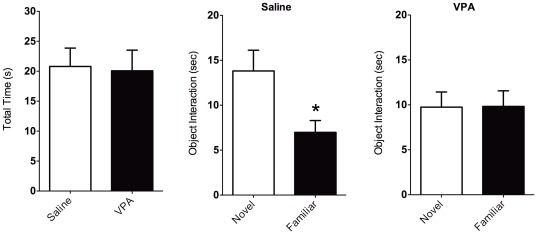
Novel object recognition in rats pre-exposed to saline and valproic acid (VPA) during gestation. Left bar graph shows total time spent exploring both objects inside a test arena. Middle bar graph shows the time saline treated rats spent exploring a novel versus a familiar object. Right bar graph shows data for VPA pre-exposed rats. While saline animals spent less time exploring a familiar object (*middle*), VPA animals spent equal amounts of time exploring each object (*right*). This was not associated with overall levels of object exploration (*left*). Data shown as mean ± standard error. Asterisks indicate significant differences p<0.05 (two tailed t-test).

**Figure 4:**
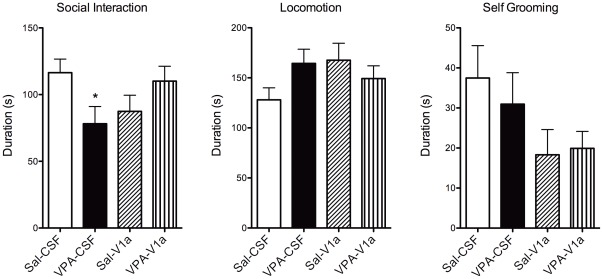
Social interactions and other behaviors in rats pre-exposed to saline (Sal) and valproic acid (VPA) during gestation. Measurements were taken immediately before setup for fMRI studies and 60 minutes after an intracerebroventricular (ICV) injection. Experiments tested whether VPA altered social behavior and neural processing, and if this involves an altered role for the V_1a_ receptor. Thus, Sal and VPA rats were subdivided into animals that were given an ICV injection of Sal or a vasopressin V_1a_ receptor antagonist. The groups are: Sal-CSF (open bar), VPA-CSF (close bar), Sal-V_1a_ (diagonal patterned), and VPA- V_1a_ (upward patterned). Data shown as mean ± standard error. VPA rats showed lower social interaction (left) and trended towards higher locomotor activity (p = 0.07). *Significantly different from Sal-Sal (p<0.05; two way ANOVA).

**Figure 5:**
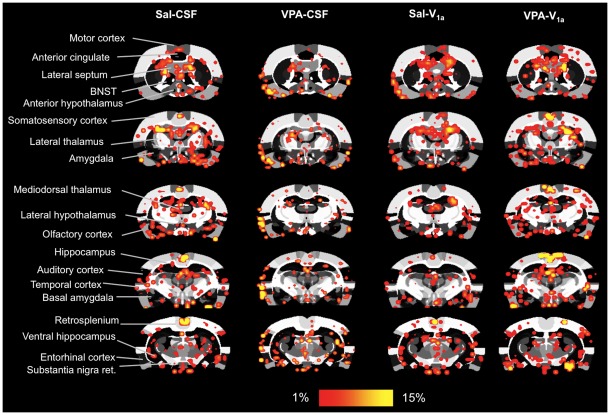
Blood oxygen level dependent (BOLD) response to a social stimulus in rats pre-exposed to saline and VPA during gestation. The study design is as explained in Figure 4. Sal and VPA rats were subdivided into animals that were given an ICV injection of Sal or a vasopressin V_1a_ receptor antagonist. The groups are: Sal-CSF (n = 10), VPA-CSF (n = 8), Sal-V_1a_ (n = 13), and VPA-V_1a_ (n = 10). An *in vivo* social stimulus juvenile was presented during scanning between repetitions 51–100, with repetitions 1–50 corresponding to baseline acquisitions (see text for details). Shown are 2D atlas maps for the different treatment conditions. BOLD maps are composites of 8–13 subject functional scans. Several ROI's included in the analysis are indicated in the far left column. Scale bar hue (red-yellow) shown at the bottom indicates the percentage change (from 1–15%) for areas of increased activity.

**Figure 6:**
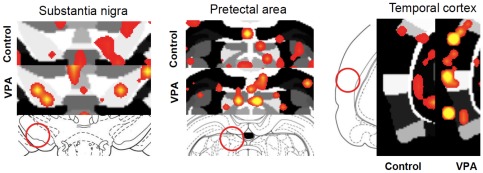
Social stimulus-induced changes in BOLD in rats pre-exposed to saline (Sal) and VPA during gestation. The study design is as explained in Figures 4 and 5. An *in vivo* social stimulus juvenile was presented during scanning between repetitions 51–100, with repetitions 1–50 corresponding to baseline acquisitions (see text for details). Shown are 2D atlas maps for two treatment conditions (Sal and VPA). Close ups highlight several ROI's of saline control and VPA treated animal's. Scale bar hue (red-yellow) shown at the bottom indicates the percentage change (from 1–15%) for areas of increased activity. Note predominant yellow hue (indicative of higher activity) pixels in VPA-CSF compared to saline (Sal-CSF).

**Figure 7:**
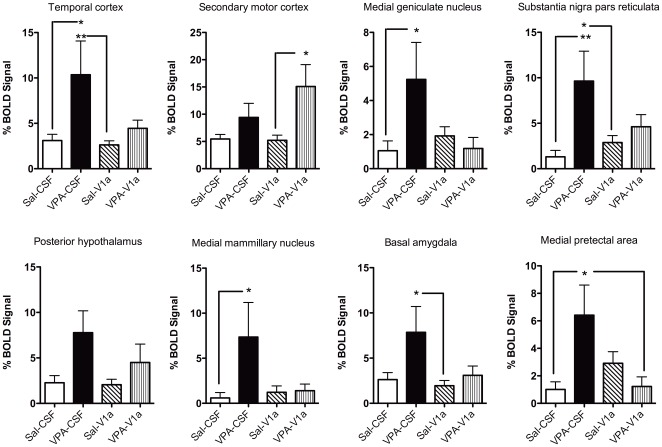
Social stimulus-induced changes in BOLD in rats pre-exposed to saline (Sal) and VPA during gestation. The study design is as explained in Figures 4 and 5. Sal and VPA rats were subdivided into animals that were given an ICV injection of Sal or a vasopressin V_1a_ receptor antagonist. The groups are: Sal-CSF (open bar), VPA-CSF (close bar), Sal-V_1a_ (diagonal patterned), and VPA-V_1a_ (upward patterned). The percent changes correspond to the averaged timecourse for the stimulus epoch. Graphs show percent change in BOLD across groups for various ROI's. Data shown as mean ± standard error. Asterisks and lines indicate specific posthoc comparisons with * p<0.05 and **p<0.01 (two way ANOVA).

**Figure 8:**
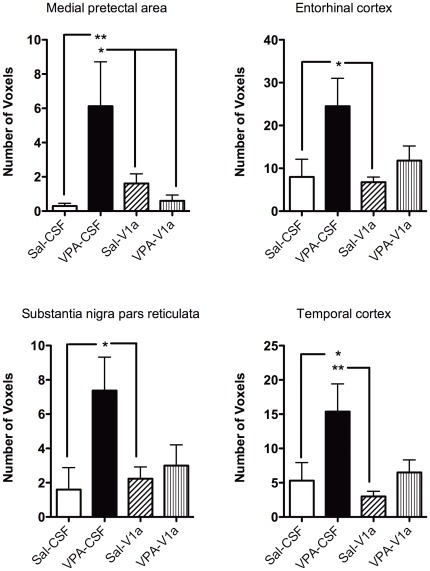
Number of voxels in rats pre-exposed to saline (Sal) and VPA during gestation. The study design is as explained in Figures 4 and 5. Sal and VPA rats were subdivided into animals that were given an ICV injection of Sal or a vasopressin V_1a_ receptor antagonist. The groups are: Sal-CSF (open bar), VPA-CSF (close bar), Sal-V_1a_ (diagonal patterned), and VPA-V_1a_ (upward patterned). Data correspond to voxel counts per ROI that showed significant BOLD signal increases. Similar to Figure 6, VPA-CSF rats show greater BOLD activation (number of voxels) than Sal-CSF. The V_1a_ antagonist reduces this effect in VPA rats. Data shown as mean ± standard error. Asterisks and lines indicate specific posthoc comparisons with * p<0.05 and **p<0.01 (two way ANOVA).

**Figure 9:**
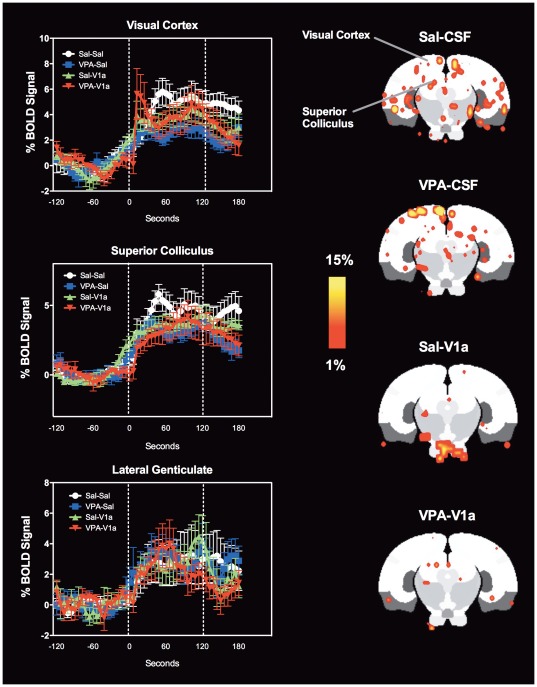
Visual system BOLD activation in response to 2 Hz flashing LED in rats pre-exposed to saline and VPA during gestation. The study design is as explained in Figures 4 and 5. Sal and VPA rats were subdivided into animals that were given an ICV injection of Sal or a vasopressin V1a receptor antagonist. Left column shows BOLD signal time courses for the visual cortex, superior colliculus and lateral genticulate nucleus. The groups are: Sal-CSF (white circles), VPA-CSF (blue squares), Sal-V_1a_ (green triangles), and VPA-V_1a_ (red inverted triangles). Data shown as mean ± standard error. BOLD signal changes in Sal-CSF rats (white circles) were greater in the visual cortex than VPA-CSF rats (p<0.05, repeated measures ANOVA). Shown on the right are 2D maps at the level of the visual cortex. Scale bar hue (red-yellow) indicates the percentage change (from 1–15%) for areas of increased activity.

**Figure 10:**
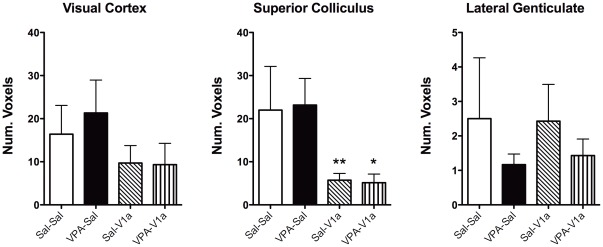
Number of voxels in visual pathway regions of rats pre-exposed to saline and VPA during gestation. The study design is as explained in Figures 4 and 5. Sal and VPA rats were subdivided into animals that were given an ICV injection of Sal or a vasopressin V_1a_ receptor antagonist. The groups are: Sal-CSF (open bar), VPA-CSF (close bar), Sal-V_1a_ (diagonal patterned), and VPA-V_1a_ (upward patterned). VPA treatment did not have the same effect on the visual stimulus as with the social stimulus (VPA-CSF versus Sal-CSF). V1a receptor blockade reduced voxels in superior colliculus, an area containing a moderate density of V_1a_ receptor mRNA [19], in both Sal and VPA rats. Data shown as mean ± standard error. Data shown as mean ± standard error. Asterisks indicate specific posthoc comparisons with * p<0.05 and **p<0.01 (two way ANOVA).

The goal of the present study was to begin to characterize behavioral and neural alterations relevant to social interactions in animals that were gestationally exposure to VPA. The dose of VPA and timing of the treatment was based on previous work showing behavioral alterations resembling features of ASD [10]. Putative changes in social neural processing and altered social behavior was the focus of the research. It was anticipated that any changes in social behavior and brain function would be mediated in part through an altered functional role of the AVP V_1a_ receptor subtype. Figures 1,2,3,4 report data for behavioral effects of gestation VPA exposure, while Figures 5,6,7,8,9,10 report functional MRI results.

### Pup weights and other physical measures of newborn pups

VPA and saline treated rats did not differ in their overall weights (**Figure S1**). Both groups had comparable weight gain. Age of eye opening and anogenital distances was similar between VPA and saline pretreated rats. These data suggest that the dose and timing of gestational VPA treatment did not produce overt structural and anatomical modifications that are consistent with its role as a teratogen.

### Pup ultrasonic vocalizations (USV's) in the early-to-mid neonatal period (PND5 and PND11)

We observed variations in USV call patterns that were dependent on both gestational VPA treatment and postnatal day in which the measurements were taken. Data are shown in **Figure 1**. Call patterns in rodents have complex frequency and spectral features. We observed various stereotypical waveform patterns on spectrograms that were categorized for analysis. A large majority of USV's were observed within the 40 kHz range, although there were calls emitted at higher >50 KHz and at lower frequencies (∼30 kHz). In addition, eight USV types were easily identifiable. These included complex, two-syllable, frequency steps, flat, chevron, short, downward and upward types (**Figure 1**). Criteria for their classification are provided in **Table S1**. The upper row of graphs in Figure 1 shows that complex and frequency step types increase in quantity on postnatal day (PND) 11 compared with PND5 (complex p = 0.0003 and frequency steps p = 0.0002, Kruskall-Wallis ANOVA). VPA treatment did not alter this increase. However, VPA pretreatment resulted in a higher level of complex USV calls on PND5 (Figure 1; VPA D5 vs. SAL D5, p = 0.009, Kruskall-Wallis ANOVA). The bottom row of graphs shows that VPA rats had a higher quantity of downward type USV calls (VPA D5 vs SAL D5; p = 0.006, Kruskall-Wallis ANOVA). This difference was not present by PND11 (**Figure 1** downward type graph). No additional differences were found in other USV call types. The USV data therefore indicates that specific types of pup calls, which might reflect early differences in social communication, are altered by VPA treatment.

### Adolescent play-fighting and social behavior (PND35–40)

VPA treated rats showed greater levels of general activity (locomotor activity) than saline controls (t-test t_20_ = 2.1, p = 0.03; **Figure 2**), but no differences in self-grooming. VPA rats also showed reduced social interactions with the juvenile intruder rat (t-test t_20_ = 2.1, p = 0.04). This included approaching, sniffing, grooming the intruder. Grouped play fighting showed no differences between the treatment conditions (**Figure 2**). However, upon categorization we observed reduced movements over the nape of the intruder by the resident test animal (t-test t_20_ = 2.1, p = 0.04; **Figure S2**). These data provide evidence that VPA treatment affects social behavior and play fighting during adolescence.

### Novel object recognition (NOR) test

**Figure 3** shows the results for a novel object recognition test. This was included in the battery of tests to confirm whether cognitive changes occur with gestational VPA exposure. Although saline and VPA animals did not show differences in general interactions with the objects inside the cage, saline animals spent significantly more time exploring (sniffing, establishing contact, exploring) the novel versus familiar object (t-test t_11_ = 2.6, p = 0.04). VPA animals spent an equal amount of time approaching both objects (novel and familiar). Thus, in addition to early life deficits in social behavior, play fighting and altered USV calls, VPA exposed rats also show signs of cognitive deficits.

### Vasopressin V_1a_ modulation of social interactions in adulthood

**Figure 4** shows the results of a social interaction test carried out prior to fMRI studies. Two-way ANOVA revealed significant main effects of both prenatal and pre-imaging treatments on social interactions (F_1,30_ = 6.5,p = 0.01) and grooming (F_1,30_ = 5.0, p = 0.03), but not locomotor activity (F_1,30_ = 3.5, p = 0.06). VPA rats (PND60–65) showed significantly lower interactions with a juvenile test rat than saline animals (Bonferroni posthoc test, p = 0.04). However, animals that received an ICV infusion of the V_1a_ antagonist did not show a similar effect (**Figure 4**). A word of caution here is warranted, however. The animals were tested almost immediately following intracerebral cannula placement; therefore the results could vary if tested following a recovery period.

### fMRI of social neural processing

Functional images were segmented into 101 regions of interest (ROI's) for the analysis. Coronal slices (1 mm thickness each) covered the rostral-caudal extent from the most rostral aspect of the prefrontal cortex and anterior olfactory nucleus-olfactory bulb junction to the midbrain/visual cortical areas. Saline (Sal) and VPA rats were subdivided into animals that were given an ICV injection of Sal or an AVP V_1a_ receptor antagonist. The groups are: Sal-CSF (n = 10), VPA-CSF (n = 8), Sal-V_1a_ (n = 13), and VPA-V_1a_ (n = 10). Note the different vehicles used for prenatal treatments (Sal) and pre-imaging V_1a_ blockade (artificial cerebrospinal fluid, CSF). An *in vivo* social stimulus juvenile was presented during scanning between repetitions 51–100, with repetitions 1–50 corresponding to baseline acquisitions (see text for details). Two-dimensional coronal maps are shown in **Figure 5** for the four treatment conditions. The maps correspond to averaged composites across all subjects within each treatment group. Therefore, areas showing increases in the blood oxygen level dependent (BOLD) signal are averages of well-aligned structures. There is significantly increased BOLD in anterior hippocampal, temporal cortical medial thalamic and midbrain regions upon presentation of the juvenile male during scanning. The hue of the red-yellow pixilated areas indicates the extent or magnitude of activation within the indicated regions, whereas the spread of the voxels across the image plane indicates the volume of activated tissue (voxels). The temporal cortex, substantia nigra, medial pretectal area, ventral hippocampal areas show greater magnitude BOLD signal responses in the VPA-saline group compared to the saline control animals (**Figure 6**). Amygdala-hypothalamic areas show greater volume of activation in saline controls presented with the social stimulus juvenile male. This pattern of activation was reduced in VPA animals receiving the ICV antagonist of the V_1a_ receptor (**Figure 5**).

Statistical analyses between the distinct treatment groups revealed a subset of regions that showed greatly increased BOLD activity (% BOLD signal) in VPA compared to saline controls (**Figure 7**). In many of these regions, blockade of V_1a_ receptors with the antagonist resulted in a reduction of VPA's effect on brain BOLD responses (**Figures 7** **and** **8**). In the following regions there was a main significant effect of prenatal treatment and/or interactions between prenatal and preimaging treatments. These included the temporal cortex (F_3,37_ = 8.1, p = 0.006), medial genticulate nucleus (F_3,37_ = 5.9, p = 0.02), substantia nigra (F_3,37_ = 10.4, p = 0.002), posterior hypothalamus (F_3,37_ = 7.2, p = 0.01), medial mammillary nucleus (F_3,37_ = 4.5, p = 0.04), and medial pretectal area (F_3,37_ = 10.3, p = 0.002). **Figure 7** rows show the quantitative data for several of the regions showing the greater magnitude BOLD responses. There are several areas that showed a different pattern of activation. These included the secondary motor cortex, where VPA-V_1a_ animals showed a greater BOLD response than saline-V_1a_ rats (F_3,37_ = 8.6, p = 0.005). Basal amygdala BOLD activation was significantly greater in VPA-CSF than saline-V_1a_ animals (F_3,37_ = 5.7, p = 0.02). We also analyzed the volume of activation with ROI's and observed that there is a similar pattern (VPA-associated increases in BOLD and reduction of this effect with V_1a_ receptor antagonism). These included the medial pretectal area (F_3,37_ = 4.8, p = 0.03), entorhinal cortex (F_3,37_ = 8.2, p = 0.006), substantia nigra (F_3,37_ = 6.9, p = 0.01) and temporal cortex (F_3,37_ = 8.7, p = 0.005) (**Figure 8**). The neural processing of a social stimulus, under the present experimental conditions, appears to be altered in VPA animals. Most areas show a robust increase in BOLD response to a social stimulus (**Figures 7** **,** **8**). However, blockade of the AVP V_1a_ receptor reduces this effect in VPA exposed, but not saline exposed, rats. This, our data suggest that the alterations in BOLD activity may in part be due to changes in AVP neurotransmission.

### fMRI of primary sensory (visual) processing

Given the results on the novel object recognition test (where animals treated with VPA showed diminished novel object/familiar object distinction), we tested the BOLD response of the rat visual system to the presentation of a switch operated flashing (2 Hz) light emitting diode placed approximately 2.5′ away from the center of the bore, but directly within the animal's visual plane. Results are shown in **Figures 9** **,** **10**. There is clear activation of areas of the visual system, including the visual cortex, lateral genticulate nucleus and superior colliculus. A close inspection of the % BOLD time courses revealed a greater BOLD activation in saline controls compared with VPA animals (one-way repeated measures ANOVA comparing saline-CSF and VPA-CSF animals, *F*_1, 550_ = 6.3, p = 0.04; no difference observed with VPA-V_1a_ p = 0.3). This was observed in the visual cortex but not the superior colliculus (p = 0.1) or lateral genticulate nucleus (p = 0.8) (**Figure 9**). Treatment with the V_1a_ antagonist had no effect on percent change in BOLD over time with light stimulation (**Figure 9**). We also analyzed the number of voxels showing increases in BOLD (volume of BOLD activation). The superior colliculus showed a reduced number of voxels in V_1a_ antagonist groups (both saline and VPA rats) compared to their counterparts (p = 0.004) (**Figure 10**). However, the effect of the V_1a_ antagonist was not selective for the VPA animals alone. Contrary to our findings with the social stimulus, there is a reduced (instead of increased) BOLD activity in response to light in primary visual cortex of VPA pre-exposed animals. This remained unaffected by the V_1a_ antagonist (**Figures 9** **and** **10**). The V_1a_ antagonist reduced overall light induced BOLD activity in the superior colliculus (**Figure 10**), a region known to contain mRNA transcripts for the V_1a_ receptor [19].

## Discussion

The behavioral data show developmental defects associated with gestational VPA exposure on cognition, locomotion and social interactions that is consistent with previous work [10]. Changes in USV patterns were observed between PND5 and PND11 that were independent of the VPA treatment. USV calls vary between developmental time points, and partly reflect underlying neurobiological modifications that need further investigation [21]. Complex and frequency step USV calls were greater in PND11 than PND5. Other call types did not change between postnatal days. VPA's effect on USV calls seemed specific for complex, downward, and chevron types. Alteration of these calls were observed on PND5, but were not present by PND11. This effect might be related to postnatal changes in synaptic activity and neuronal excitability reported recently [22]. USV calls are therefore modified and this could potentially reflect underlying alterations in the brain as a result of gestational VPA exposure. However, these changes appear transitory within the postnatal developmental period. Play fighting during adolescence was not significantly affected in our hands, although we did observe a reduction in attack-like movements to the nape. However, general social interactions monitored during adolescence and adulthood was lower in VPA animals, while locomotion increased above control levels. Collectively, the behavioral data from early postnatal, adolescence and adulthood support an effect of VPA on social behavior. These results align well with previous reports [10]. Given these findings, we hypothesized that VPA exposure effects on social behavior may also be accompanied to some extent by cognitive deficits [10]. We tested a group of animals on a simple cognitive task to determine whether a form of short-term memory, in addition to social behavior, was affected by prenatal VPA. We observed that VPA rats show a defect in NOR, as demonstrated by a lack of significant difference between the time spent exploring a novel object versus a previously encountered object. Overall, our data support an effect of prenatal VPA on social behavior that was further examined in adult rats that were imaged for their response to a social stimulus.

Our neuroimaging data also provides evidence of a lasting effect of prenatal VPA on social neural processing. VPA-exposed rats show significantly greater BOLD signal responses to a stimulus juvenile rat than saline control animals. Even though the observed behavioral changes suggest a deficit, the neural responses to a social stimulus in VPA rodents were of greater magnitude. This was observed in several cortical and subcortical limbic regions, including temporal, secondary motor and entorhinal cortices, basal region of the amygdala, medial genticulate, posterior hypothalamus and mammillary nuclei, and the substantia nigra pars reticulata. We also provided a primary sensory stimulus to study three brain regions of the visual pathway to examine whether differences in social neural processing could be attributable to changes in primary sensory processing, albeit through a specific sensory system. Interestingly, we observed that the greater activation found with the social stimulus was not observed with the visual stimulus. Instead, the visual cortex of VPA rats showed a lower percentage BOLD signal responses that perhaps occurs as a result of lower neural activity in this region. The effects of VPA on primary sensory systems were thus distinct from subcortical and higher order cortical processing of a socially salient stimulus. While the complex social stimulus generates a greater BOLD activation in VPA rats compared with controls, the primary sensory stimulus has an opposite outcome, at least in the visual cortex.

In order to examine whether VPA-induced effects on social neural processing could be associated with changes in functionality activity through the AVP V_1a_ receptor, we included subgroups of animals that received an ICV injection of an antagonist for this receptor. A reduction in BOLD activity in these regions was observed with V_1a_ blockade. The antagonist on its own had no effect on the pattern of brain activation shown in control animals. Thus, the effects of V_1a_ blockade seemed to be selective for the VPA pretreated animals, supporting an effect of prenatal exposure on vasopressin neurotransmission. Moreover, the effect of the antagonist was not found to affect primary sensory processing (visual). Therefore the actions of the antagonist could be interpreted as occurring in conjunction with VPA-induced deficits in higher order processing but not primary visual processing. However, it is important to point out that, as a stimulus, the juvenile rat presentation paradigm could evoke various forms of sensory activation and not just visual. In fact, olfactory stimulation could also account for the differences in the effects of the antagonist. We did not include the olfactory bulb in our present MR scanning procedure. The purpose, however, of the alternative light stimulation was to simply test sensory processing without the influence of interacting olfactory effects, which would clearly occur as a result of the presentation of a social stimulus before or after a scent. Presenting a scent after presentation of the social stimulus would likely have had a competing effect with unintended consequences that we avoided during scanning by simply using a visual stimulus.

Our data are in agreement with the work of Markram and colleagues [15], [18], [23], [24], [25]. They have repeatedly shown in series of elegant in vitro electrophysiological studies that areas of the cortex (somatosensory and prefrontal, for example) show a greater degree of local but not distant synaptic connectivity in VPA exposed rats compared to controls [18]. These synapses are more abundant in quantity but show weakened stimulated membrane conductance [18]. Interestingly, and despite the weaker excitatory currents, the cortical synapses of VPA rats show prolonged long-term potentiation (LTP) [24]. Both the greater number of local synapses and the enhanced LTP could represent biological mechanisms that underlie the hyperresponsive BOLD activity we observe in VPA animals in the present study. Their results point to altered cortical wiring with VPA exposure during gestation, which may affect cognition, emotionality and social behavior during the lifespan of the rodents [15]. Consistent with this work, we observed here that VPA animals show greater BOLD activation in response to a social stimulus. From the composite 2D maps in **Figure 6** it is possible to observe greater magnitudes of BOLD with reduced volume of activation. Although not quantitatively examined, this could represent a pattern that is consistent with the findings of a greater degree of local but not distant synaptic connectivity in VPA exposed rats [18]. Our results using the light stimulus could indicate however that the observed pattern of activity is not uniform across the brain (**Figure 9**).

Blocking V_1a_ receptors reduced the BOLD responses to a social stimulus in VPA exposed animals only. We do not know whether the number of receptors, the release of AVP, or the number of fibers containing AVP accounts for the increased activity. The areas that showed increased effects of VPA and blockade by the AVP antagonist were not the same as those studied by Markram and colleagues, which were the prefrontal cortex and somatosensory cortex [18], [25]. Our experimental model did not appear to recruit these regions. But, it could be that the synaptic mechanisms described in their work involve other areas of the brain, including those we observe here to be affected by the prenatal exposure. At a first glance, the brain regions showing significant effects of VPA and V_1a_ receptor blockade are difficult to piece together into an actual neural network and an attempt to do so is speculative. However, there is strong evidence these areas are known to play roles in social cognition, arousal, attention and motor function, all behavioral functions affected in ASD. Moreover, the effects of VPA in the basal region of the amygdala, temporal cortex, medial genticulate, entorhinal cortex, brings to mind part of the neural circuitry of contextual fear conditioning [26]. These areas are perhaps responding to the experimental conditions in these animals, an effect not seen as robustly in controls. It also could be that the greater neural activation in response to a social stimulus is accompanied by high fear levels [15]. Therefore, sounds associated with a social interaction are perhaps activating regions involved in fear reactivity [15]. The effects of V_1a_ blockade observed in other regions such as the temporal cortex, motor cortex, substantia nigra are likely to be indirect effects of receptors located in distal afferent sites. Although there is some scant expression of V_1a_ messenger across various cortical areas, these are in very low amounts compared to subcortical expression [19]. The heteregenous expression of V_1a_ mRNA in forebrain and midbrain regions can account for the results presented here. Some of these include the posterior hypothalamus/mammillary nuclei, ventral hippocampus, lateral septum, and regions of the basal ganglia [19]. However, a study to look at the expression of AVP or the V_1a_ messenger in the cortex and other subcortical areas of VPA treated animals would be needed to determine whether AVP activity or its receptor are increased.

There are several shortcomings of the study and alternative explanations that should be noted. First and foremost, the in vivo presentation of stimuli to the rats that was imaged presents a highly complex picture of brain processing that is limited in its interpretability. Although it provides a real world examination of general patterns of brain activity during a social encounter, it is difficult to tease out the specific sensory systems that are recruited and the selectivity of the neural pathways involved. Also, while the acclimatization minimizes motion, the stress associated with restraint is not entirely removed. In addition to this, the animal that is used for presentation is likely to also suffer from stress and distress, which could lead to the emission of vocalizations detected by the imaged rat. Therefore, the neural activity measured in response to social stimulus presentation could be associated with recognition of the social stimulus, but also can reflect the response to emitted 22 kHz distress calls on the part of the stimulus juvenile rat, odors associated with the stimulus rat and perhaps visual stimulation upon presentation of the juvenile. Despite this, the neural processing, although complex, does involve a recognized social stimulus, which was a goal of the study design. An adequate control for the social stimulus, with all of its intricate properties was not used. This is actually quite difficult since the stimulus not only presents odors, but also sound and visual stimulation. Future work should take this into consideration and specific socially relevant odors for rats (urine as an unconditioned odor or an alternative conditioned odor) could be appropriate. With regards to the imaging, there is sufficient evidence that the magnitude of the BOLD signal is associated with changes in baseline bloodflow, as well as neural activity [27]. Reductions in basal blood flow allows for greater magnitude BOLD responses. Similarly, a reduction in basal neuronal activity is permissive of greater magnitude increases in activity, and oxygen metabolism [28], [29]. Therefore, the actions of the V_1a_ antagonist could arguably be associated with reductions in basal neuronal excitability as well as blood flow. However, in both of these scenarios, the effects of the antagonist appear specific for the VPA treatment.

Another concern of the present work relates to the behavioral assessments after cannulation. We carried out a brief behavioral test after placement of the intracerebral cannula and injection of the V_1a_ antagonist. The conditions for testing were therefore not optimal for social recognition testing. The same could be said of the fMRI studies 90 minutes post cannulation and injection. As stated above, the fMRI conditions could reflect distressful conditions that may hinder clear interpretations solely based on the processing of social stimuli. Also, the fact that the brief NOR test results show reduced cognitive functionality could indicate that there are also memory impairments or deficits in sensory processing in addition to impairments in social neural processing. This is partly supported by our findings in **Figure 9** **,** **10**. One final caveat is that a neurochemical correlate clearly showing an association between the altered role of V_1a_ vasopressin neurotransmission was not determined. Future studies will address this by immunostaining for vasopressin or mRNA studies for the V_1a_ receptor, which could be informative when compared to the present imaging results.

As expected, the role of V_1a_ receptors in modulating socially relevant neural circuits was influenced by prenatal VPA treatment. This is consistent with the role of the AVP receptor subtype in social behaviors and could be one substrate affected by VPA. Further data using neurochemical techniques are needed to confirm whether AVP or its receptors or both are affected. Polymorphisms of the V_1a_ receptor gene within its promoter site, RS3, have been associated with altruistic behavior [30], ASD [31] and pair bonding behavior in humans [4]. The RS3 long form of the promoter-repeat sequence is associated with higher altruistic behavior and greater hippocampal levels of the V_1a_ receptor mRNA than the RS2 short sequence [30]. The results in humans partially replicate work in prairie voles [32] by showing that these microsatellite repeats in V_1a_ 5′-flanking region are functionally related to social bonding in male voles [33]. Knockout mice lacking the V_1a_ receptor gene show deficits in social memory tests and reduced anxiety [3], [34]. Individual variations in the expression of AVP also underlie the expression of anxiety related behaviors in rodents [35], [36]. The role of V_1a_ receptors, and also oxytocin receptors, in the regulation of emotion, cognition and social behavior merits further investigation, within the context of the neurobiology of ASD.

Our results show a greater BOLD response to a social stimulus in the VPA rat. This is blocked by the AVP V_1a_ receptor antagonist. Therefore, the greater ‘sensitivity’ to social stimuli could in part be mediated by excitatory AVP neurotransmission. The experiments summarized here leaves important unanswered questions that will be pursued in subsequent research. For instance what is the precise in utero mechanism through which VPA affects AVP neurotransmission and neural circuits of social behavior? On possible mechanism may involve one of VPA's intracellular targets. VPA directly binds to *HDAC1* to produce inhibitory effects similar to butyrate. The observed actions of VPA seemed to be mediated within a finite window of action centered on the period of neural tube closure. Much like butyrate, VPA binds reversibly to HDAC enzymes and inhibit their action, thereby potentially leading to a transient relaxed chromatin state that favors gene expression. Our present results, far from providing conclusive mechanisms, provide potential directions in which to confirm the effects of VPA on social neural circuits, whether the treatment has lasting effects on the vasopressin system and finally whether specific chromatin modifications or other molecular actions in utero occur during development of these systems.

## Materials and Methods

### Subjects

Long-Evans female rats (175–225 g) were purchased from Charles River Laboratories (Wilmington, MA). Females were housed in pairs in a temperature and humidity controlled room and maintained on a 12 hr light-dark cycle (12L∶12D, lights off at 1900 hr). All experiments were carried out between 0900–1700 hrs. Females were single housed during their pregnancy and with their litter during the postpartum period. Home cages consisted of hanging plastic microisolater cages of standard dimensions with woodchip bedding. Water and Purina rat chow were provided *ad lib*. The general design of the study involved the treatment of timed pregnant rats with saline or VPA during midgestation and experimental analysis of behavior during early development, adolescence and adulthood. Functional MRI studies are performed in control and treated animals during adulthood to examine alterations in brain regions responsive to social stimuli. Behavioral assessments were done to confirm whether prenatal treatment with VPA affects social behavior across various time points across the lifespan of the animal.

Procedures involving research animals were conducted in accordance with the guidelines published in the Guide for the Care and Use of Laboratory Animals and adhere to the Office of Laboratory Animal Welfare of the National Institutes of Health and the American Association for Laboratory Animal Science. The Institutional Animal Care and Use Committee at Northeastern University provided prior approval of the protocols used in this study.

### Drugs

VPA (disodium salt form) and the AVP V_1a_ antagonist [β-Mercapto-β,β-cyclopentamethylenepropionyl^1^, O-me-Tyr^2^, Arg^8^]-Vasopressin were purchased from Sigma-Aldrich Chemical Co. (St. Louis, MO). VPA was prepared by dissolving it in 0.9% sterile physiological saline solution at a concentration of 250 mg/mL and was injected intraperitoneal (IP) at a dose of 600 mg/kg [10]. Control animals received saline injections. The V_1a_ antagonist was dissolved in artificial cerebrospinal fluid (aCSF; Harvard Apparatus, Holliston, MA) and injected intracerebroventricularly (ICV) at a dose of 125 ng/10 µL 90 minutes before imaging experiments. We have successfully used this antagonist dose and volume in our previous work [37]. Controls rats received only 10 µl of aCSF. All injections into ventricles were made unilaterally.

### Gestational valproic acid administration

Females were housed overnight with an experienced male breeder. In order to confirm pregnancy, daily vaginal smears were examined under a light microscope each morning for the presence of sperm flagellae. The presence of spermatozoa was designated as day 1 of gestation (GD1). On GD12.5 (mid-to-late afternoon of the 12^th^ day) rats were given a single IP injection of VPA. Pregnant dams were monitored on a daily basis for changes in weight, changes in health, or in their feeding patterns. After weaning on postnatal day (PND) 23, offspring were housed in pairs of non-siblings (same treatment groups were always housed together). Pups were closely monitored for any signs of physical abnormalities. We tracked weights, anogenital distances, isolation-induced ultrasonic calls (see below) and the postnatal day of eye opening. Weights were taken on PND2, 5, 7, 13, 24 and 50. Animals were tagged on one limb or abdomen using a non-toxic marker pen to track data for each pup. Markings were refreshed on a daily basis. Only male offspring were used in these studies. A total of 23 saline and 18 VPA treated male pups were tested for vocalizations. These same animals were used in functional neuroimaging studies as adults. Separate groups of animals were used for adolescent social behavior (n = 14 saline and n = 15 VPA) and novel object recognition (NOR) tests (n = 10 saline and n = 8 VPA).

### Ultrasound vocalization (USV) recordings during the postnatal period

USV's were measured on PND 5 and 11. To induce 40-kHz separation calls, randomly selected pups were isolated from the nest for 10 min at room temperature (20.8°C to 23.2°C). They were placed inside a sound attenuation chamber (Med-Associates, St. Albans, VT). Ultrasonic vocalizations were recorded using a condenser microphone that is sensitive to frequencies from 10–250 kHz (model CM16 Avisoft Bioacoustics, Germany). This microphone is particularly sensitive to the 50 kHz frequency range. Sound detection and storage was achieved by interfacing with an UltraSoundGate USB digital-to-analog converter and amplifier (USG 116, Avisoft). A laptop PC running Avisoft RECORDER software was used to store acoustic data as .wav files for analysis of spectral features. USV's were continuously monitored using a frequency window between 10–70 kHz and spectrograms were generated online using a Fast Fourier Transform size of 256, a sampling rate of 140 kHz, 16-bit data format. Acoustic datasets were imported into SASLab Pro (Avisoft) and spectrograms were generated during a 2-minute window for the analysis. Filtering was used to reduce background noise and a trained observer manually quantified the total number of USV waveforms on spectrograms. Previously published spectral waveform features for neonatal mice and rats were used as a guide to classify the distinct USV patterns [38], [39], [40], [41], [42], [43]. These included the following eight USV call patterns: complex, two-syllables, upward, downward, chevron, short, frequency steps and flat calls. Scoring was conducted blind to the gestational treatment (**Table S1** provides the specific criteria). Examples of the USV call types are shown on **Figure 1**.

### Adolescent play fighting and social interaction test

Adolescent rats were tested inside their home cage with wood shavings covering the floor of the cage. All testing was done during the light phase. We analyzed play fighting, time spent interacting with a cage intruder of the same strain, age, sex and weight, and general grooming activity within the home cage. Only subject data were analyzed for a given session. Color markings on the scruff of the neck were used to distinguish subjects from intruders. Intruders were used more than once. All behavior was video recorded, imported as .avi files to a laptop PC via 1394b firewire connection using Windows Movie Maker and were analyzed using ODLog software (Macropod software). Play behavior was scored as previously reported [44]. Juvenile PND 35–40 rats were socially isolated 24 hours prior to testing. Control intruder rats were randomly selected and placed into the resident's cage (control or VPA treated) for 10 minutes and behavior recorded. On any given test trial, resident and intruder juveniles did not differ by more than 15 grams in body weight. Animals were from different litters and were not cage mates. We scored the frequency of pinning (one of the animals is laying on the floor of the test cage with the other animal standing over him), number of submissions, pulling/biting, biting on the nape of the neck. The total duration of play fighting, general social interaction (approaching, sniffing, and grooming the intruder), self-grooming and spontaneous locomotion were scored.

### Novel object recognition (NOR) test

NOR testing consisted of two phases [45]. Rats were first acclimatized to the test arena to minimize the effects of novelty stress on behavior. During the habituation phase, each rat freely explored the empty arena for 10 minutes on 3 separate days (clear Plexiglas arena 40-cm^3^ in size). All internal and external visual cues were maintained constant across all test days and the same experimenter collected data on all days. Videos were taken during the final test trials in which rats were examined for their time spent interacting with a familiar versus a novel object. The objects that were selected were clearly distinguishable by size, shape and color/contrast (but not smell) and were always placed in the same regions of the test arena during experimental sessions. Objects were washed between sessions to reduce odor effects during testing. These were adhered to the floor of the arena using 1-inch wide double-sided adhesive. During the first test trial, rats were placed into the test arena containing two identical objects. The animals remained in the cage for 10 minutes and then returned to their home cages. Object exploration was quantified as the time spent (in seconds) in direct interactions with each object in the test arena (this collectively included sniffing, contact, and facing the object while in close proximity). They were again re-tested one hour later in the same arena with one of the two prior objects (familiar object) removed from its location and replaced with an alternate non-identical object (novel object). Therefore, during the second 10-minute trial, rats were presented with a familiar and a novel object. Rats typically explore the familiar or habituated object less than a novel object. It was anticipated that animals spend more time exploring the novel object and failure to do so would represent failure to retain memory of the familiar object after 1 hour. However, this could also represent variations in visual sensory processing, which was tested during fMRI studies.

### Functional magnetic resonance imaging

Imaging the neural response to a social stimulus was carried out as an adaptation of the social recognition test whereby a juvenile male rodent is first presented in the home cage of the test animal and then reintroduced in order to examine time spent in social exploration. This enhances the ability to recognize the intruder juvenile rodent. The goal was to examine brain activity in response to a socially relevant and salient stimulus for the rats. Before imaging, rats were acclimated to restraint for 5 consecutive days as previously reported [46]. Two days after the last acclimation day, rats were imaged for their response to a social stimulus and a visual stimulus light. Functional MRI studies were carried out between PN60–65 in saline and VPA pre-exposed animals. Subgroups of animals received a central infusion of a V_1a_ receptor antagonist or vehicle (CSF) 90 minutes before imaging. Rats were prepared for imaging as previously reported [47], [48]. We used methods to deliver an acute dose of V_1a_ antagonist before fMRI, 90 minutes prior scanning, as previously reported [37], [49]. Rats were anesthetized with 2–4% isoflurane, 4% lidocaine cream applied to the scalp, the skull surface was exposed and the landmark suture Bregma located. A 26-gauge cannula of polyethylene tubing (PE-10: inner diameter 0.28 mm, outer diameter 0.61 mm) was implanted into the lateral cerebral ventricle (1 mm caudal to Bregma, 2 mm lateral to the midsagittal sinus, and 4 mm ventral to dura) and secured to the skull with Vetbond (Webster Veterinary Inc). **Figure S3** shows example cannula placements. Following the 10 µl infusion, and once animals were fully awake, they were returned to their home cage for ∼60 minutes, after which a 5-minute exposure to a juvenile male rat was done immediately prior MRI experiment preparations. During this time interactions with the juvenile test male was recorded. Animals were subdivided into subgroups as follows: prenatal saline/pre-imaging CSF (Sal-CSF, n = 10), prenatal VPA/pre-imaging CSF (VPA-CSF, n = 8), prenatal saline/pre-imaging V_1a_ blockade (Sal- V_1a_, n = 13) and prenatal VPA/pre-imaging V_1a_ blockade (VPA- V_1a_, n = 10). The unequal number of animals per group was a result of individual subjects showing excess motion artifact during scanning.

Functional imaging was performed using a T_2_-weighted fast spin echo pulse sequence with the following parameters: repetition time TR = 1562 ms, echo time TE = 7.5 ms, effective echo time TE_eff_ = 45 ms and an echo train length ETL = 16. Geometry was setup as follows: 12 slices, field of view of 28 mm, 1.0 mm thick slices with no gaps, data matrix of 64^2^ for functional scans and 256^2^ for anatomical scans (Thus, the in plane 2D pixel resolution was 438 µm^2^ for functional and 117 µm^2^ for anatomical scans). A full set of 12 coronal slices across the brain was collected at each effective repetition time of 6 seconds. During imaging sessions animals were presented with a juvenile rat 30–40 minutes after initial encounter outside the magnet. *In vivo* stimulus presentation was achieved using a clear Plexiglas cylinder fitted to the inner dimensions of the bore of the MR scanner [48], [50]. Previous studies have shown that there is minimal motion produced by the introduction of an in vivo stimulus inside the bore of the scanner [48]. Experiments were performed on a Bruker 7-Tesla magnetic resonance imager running Paravision 4.0 (Bruker BioSpin Corporation, Germany) using a rat volume transmit/surface receive radiofrequency coil system (insightMRI, Shrewsbury, MA). The entire imaging session included a 6-minute anatomical scan and two functional scans (10 minutes each) that lasted a total of about 40–50 minutes. For the social stimulus presentation, 100 repetitions (6 seconds per repetition) were collected and the stimulus male was presented in a plastic cylinder at repetition 50 [48]. To test whether general sensory processing was affected by prenatal VPA, a visual light stimulus was used (a 2 Hz flashing LED). For the nonsocial light presentation, 100 repetitions were collected and the light was turned on from repetition 50–70.

### Statistical analysis

Behavioral data in **Figure 1** was analyzed using a non-parametric Kruskall-Wallis analysis of variance (ANOVA), and **Figures 2** **,** **3** were analyzed with an unpaired t-test (with Welch's correction) comparing the means of saline and VPA treated animals (significant p<0.05). Social interaction test in **Figure 4** was analyzed using a two-way analysis of variance with gestational treatment (Saline X VPA) and pre-imaging injection (CSF X V_1a_ antagonist) as independent variables (significant p<0.05). Full details of the MRI data analysis using in-house software have been previously reported [48]. Scans were pre-screened for motion and drift using a priori criteria [48]. Drift was corrected using linear detrending. Scans showing severe translational motion that was deemed uncorrectable (>0.110 mm) were discarded. Each subject was registered to a digital atlas of the rat brain, based on a high-resolution anatomical scan of an adult-sized rat, and manually segmented following standard anatomical coordinates (**Figure S3**). Statistical *t* tests are performed on each subject within the original coordinate system. The baseline period used was 48 repetitions before and 48 repetitions after social stimulus presentation (for visual fMRI, 20 repetitions preceding the light stimulus and 20 repetitions after turning on light was used). Statistical *t* tests used a 95% confidence level, two-tailed distribution, and heteroscedastic variance assumptions. In order to provide a conservative estimate of significance, a false-positive detection-controlling algorithm is introduced into the analysis [51]. This ensures that the false-positive detection rate is below our confidence level of 5% [47]. Statistically significant pixels were assigned their percentage change values (stimulus mean minus control mean). Activated voxel numbers and percent signal changes were exported to Statistical Package for Social Sciences (SPSS) for statistical comparisons between groups. The number of voxels per ROI and their corresponding average percent change values were statistically evaluated using a 2×2 analysis of variance (ANOVA α = 0.05). Independent variables were prenatal treatment (saline X VPA) and acute treatment before imaging (CSF X V_1a_ antagonist). Posthoc tests for specific differences between treatment groups was done using Bonferroni multiple comparison test (p<0.05).

## Supporting Information

Table S1 **Classification criteria for ultrasonic calls emitted by neonatal rats.** Calls included the following eight USV call patterns: complex, two-syllables, upward, downward, chevron, short, frequency steps and flat calls. Previously published spectral waveform features for neonatal mice and rats were used as a guide to classify the distinct USV patterns [38], [39], [40], [41], [42], [43].
(DOC)

Figure S1 **Weight (in grams) gain during the postnatal period in pups exposed to saline or valproic acid (VPA) during gestation.** Data were collected at various postnatal time points (PD2, PD7, PD13, PD24). Data shown as mean ± standard error. No significant differences were noted between the groups.
(TIF)

Figure S2 **Categorized play behaviors in adolescent rats exposed to saline or valproic acid (VPA) during gestation.** Categories included attacks to the nape, pinning, submissions, pulling and biting. Data shown as mean ± standard error. Asterisks indicate significant differences p<0.05 (two tailed t-test).
(TIFF)

Figure S3 **Anatomical alignment of scans to an electronic atlas of the rat brain.** The three views show well-aligned structures. Images on the right panel show examples of cannula placements. Only animals with good placements inside the lateral ventricle were included in the study.
(TIFF)
