# Supplementary material for: Gestational Valproate Alters BOLD Activation in Response to Complex Social and Primary Sensory Stimuli
Source: PLoS One. 2012 May 17;7(5):e37313. doi: 10.1371/journal.pone.0037313 (PMC3355108; doi:10.1371/journal.pone.0037313)
Supplement: Table S1 — Classification criteria for ultrasonic calls emitted by neonatal rats. Calls included the following eight USV call patterns: complex, two-syllables, upward, downward, chevron, short, frequency steps and flat calls. Previously published spectral waveform features for neonatal mice and rats were used as a guide to classify the distinct USV patterns [38], [39], [40], [41], [42], [43]. (DOC) [file pone.0037313.s001.doc]

| 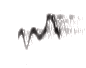 | Complex calls: one sound component containing two or more directional changes in frequency. |
| --- | --- |
| 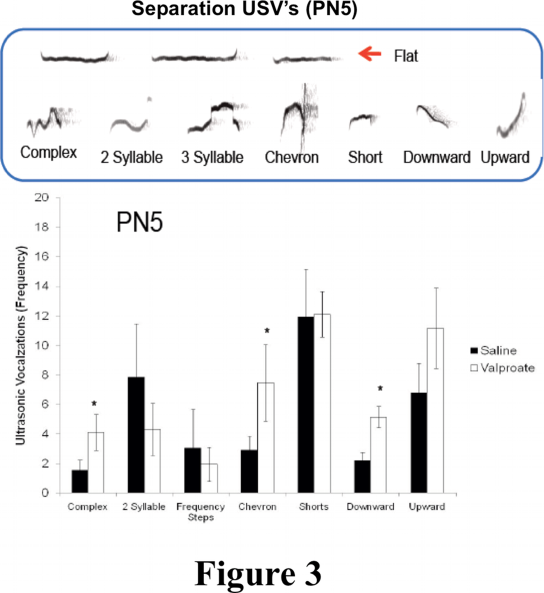 | Two-syllables: had two components, a main call (with a ﬂat or downward frequency change) with an additional short component of higher frequency. |
| 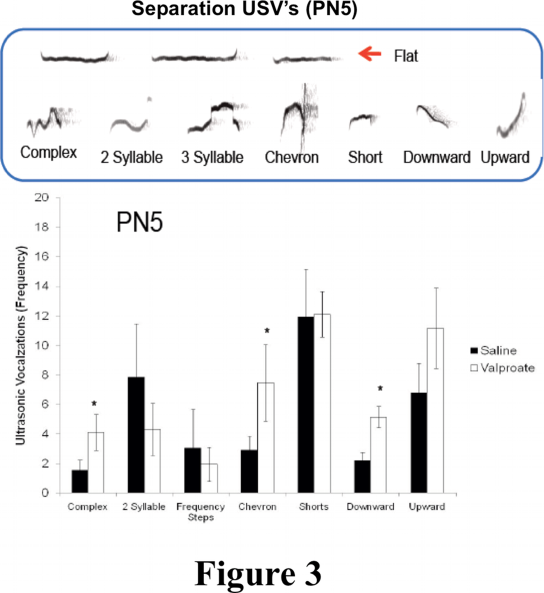 | Upward calls: showed an increase in frequency that was ≥12.5 kHz, with a terminal dominant frequency at least 6.25kHz. |
| 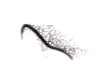 | Downward calls: showed a decrease in frequency that was ≥12.5 kHz, with a terminal dominant frequency at least 6.25 kHz. |
| 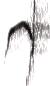 | Chevron: appeared as an inverted-U identiﬁed by an increase frequency ≥12.5 kHz followed by a decrease that was ≥6.25 kHz. |
| 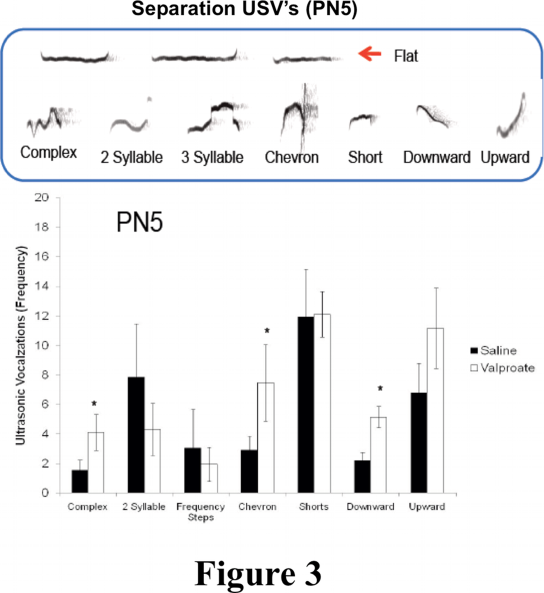 | Frequency step: had three components, a main call ﬂanked by two discontinuous lower frequency displays, with no gaps on the time scale. |
| 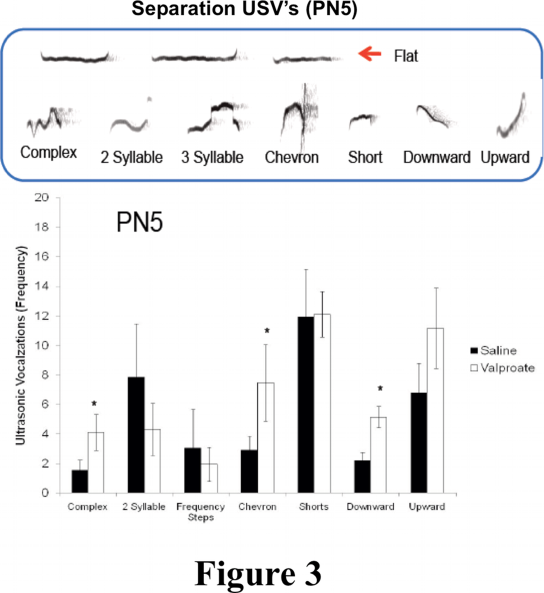 | Short calls: shorter than 5 milliseconds. |
| 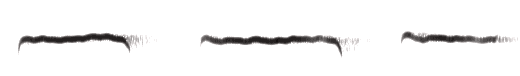 | Flat calls: constant frequency, including the initial and terminal frequency with minor changes ≤3 kHz. |

**Table S1.** Classification of pup emitted ultrasonic vocalizations.
