# Supplementary figures and images for: Gestational Valproate Alters BOLD Activation in Response to Complex Social and Primary Sensory Stimuli
Source: PLoS One. 2012 May 17;7(5):e37313. doi: 10.1371/journal.pone.0037313 (PMC3355108; doi:10.1371/journal.pone.0037313)

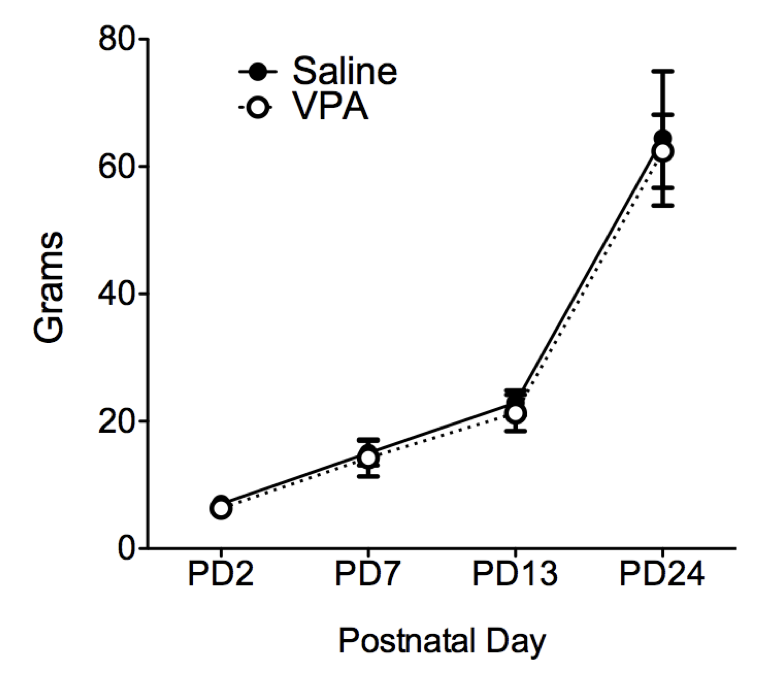

Supplement: Figure S1 — Weight (in grams) gain during the postnatal period in pups exposed to saline or valproic acid (VPA) during gestation. Data were collected at various postnatal time points (PD2, PD7, PD13, PD24). Data shown as mean ± standard error. No significant differences were noted between the groups. (TIF) [file pone.0037313.s002.tif]

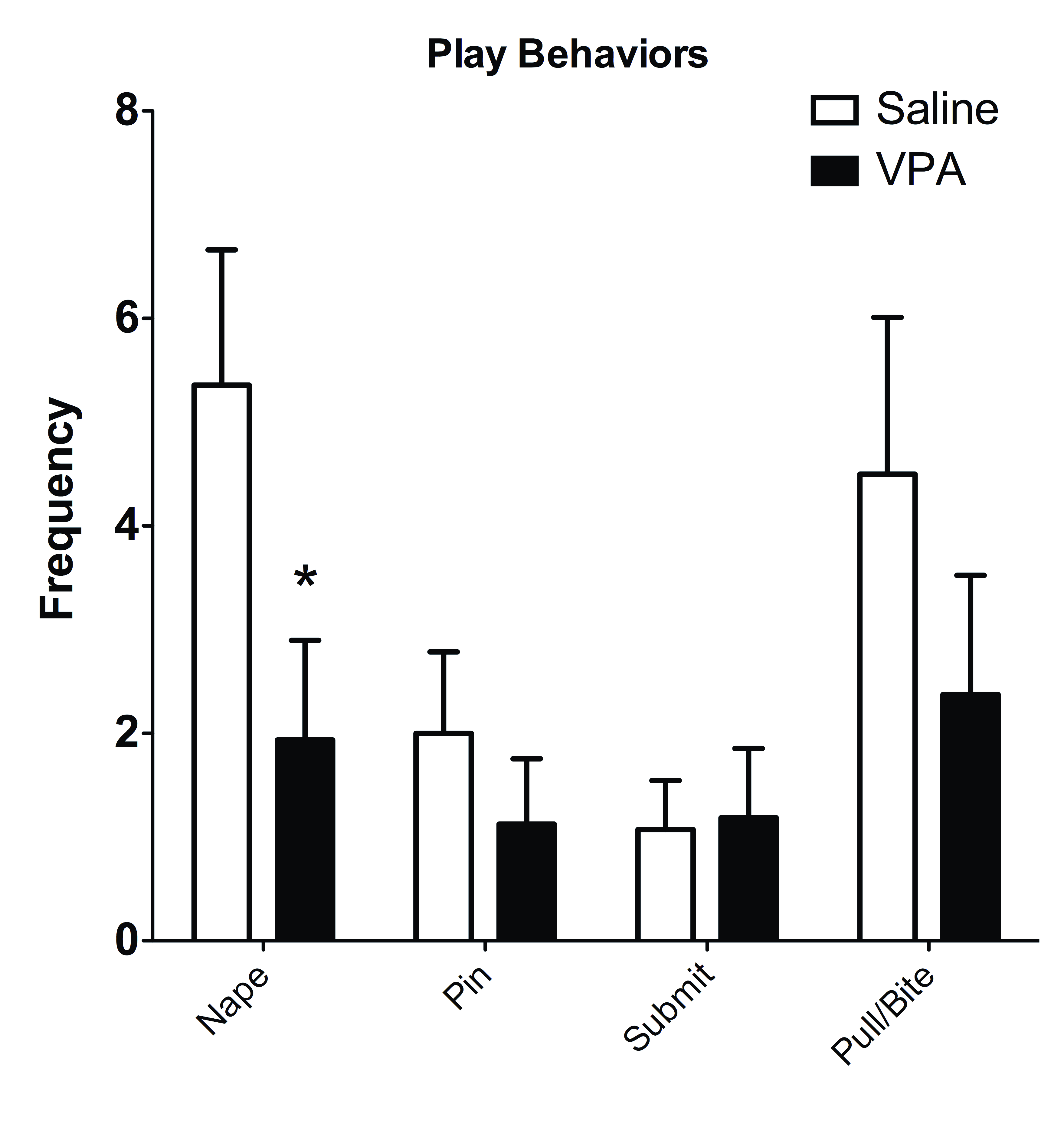

Supplement: Figure S2 — Categorized play behaviors in adolescent rats exposed to saline or valproic acid (VPA) during gestation. Categories included attacks to the nape, pinning, submissions, pulling and biting. Data shown as mean ± standard error. Asterisks indicate significant differences p<0.05 (two tailed t-test). (TIFF) [file pone.0037313.s003.tiff]

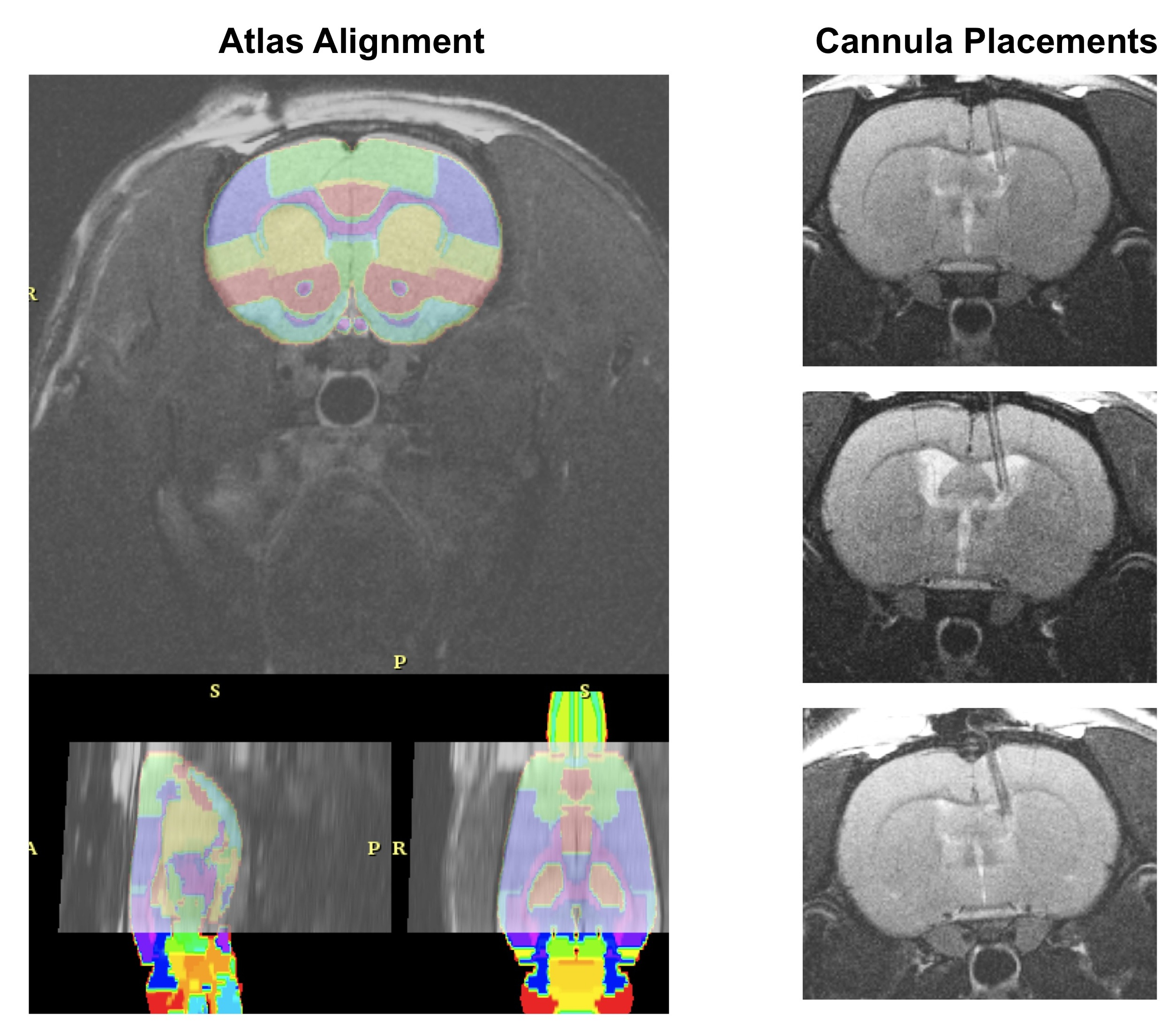

Supplement: Figure S3 — Anatomical alignment of scans to an electronic atlas of the rat brain. The three views show well-aligned structures. Images on the right panel show examples of cannula placements. Only animals with good placements inside the lateral ventricle were included in the study. (TIFF) [file pone.0037313.s004.tiff]
